# Supplementary material for: Host immunity modulates the efficacy of microbiota transplantation for treatment of Clostridioides difficile infection
Source: Nat Commun. 2021 Feb 2;12:755. doi: 10.1038/s41467-020-20793-x (PMC7854624; doi:10.1038/s41467-020-20793-x)
Supplement: Supplementary file 1 — Supplementary Information [file 41467_2020_20793_MOESM1_ESM.pdf]

Supplementary Figure 1

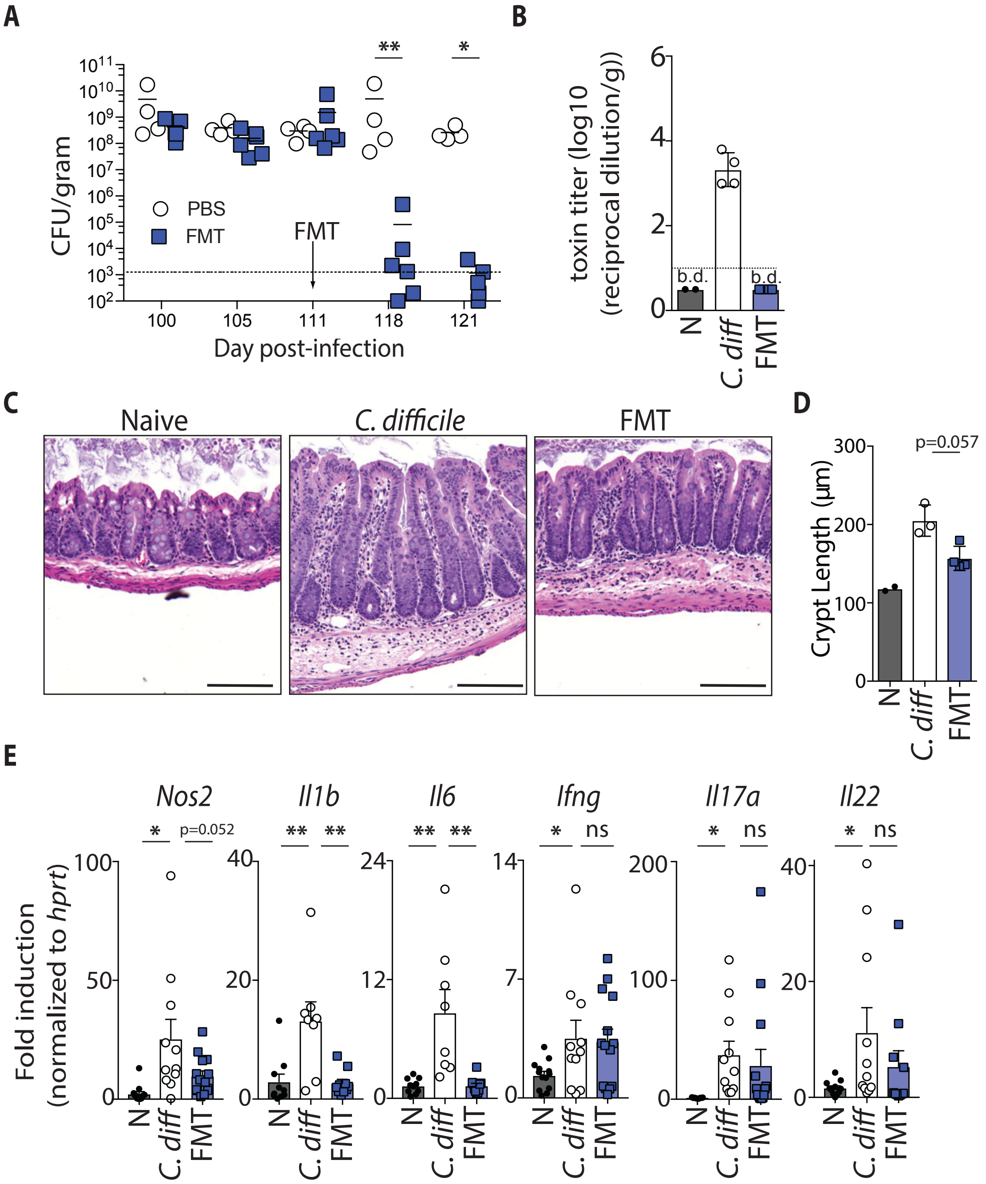

Supplementary Figure 1. Fecal Microbiota Transplantation (FMT) resolves C. difficile infec-tion and ameliorates chronic intestinal inflammation. (A) C. difficile burden in fecal pellets of C57BL/6 mice following FMT (n=6) or PBS (n=4) treatment. Statistical significance was calculat-ed by a two-sided unpaired Mann-Whitney test; *p=0.016, **p=0.0095. (B-E) Mice were sacrificed at day 10 post-FMT or PBS treatment and compared to naïve, C57BL/6 mice. (B) Toxin B levels in the cecal content. Data are presented as mean values ± SEM. (C) H&E stained cecal tissue sections. Scale bar = 100 m. Images representative of two independent experiments. (D) Mean crypt length. n=2 Naïve; n=3 C. diff; n=4 FMT groups. Data is representative of 2 indepen-dent experiments. Statistical significance was calculated by a two-sided unpaired Mann-Whit-ney test. Data are presented as mean values ± SEM. (E) mRNA gene expression of proinflamma-tory immune response genes in whole colon tissue quantified by qRT-PCR. Gene expression relative to naïve, C57BL/6 mice and normalized to Hprt. For Nos2, Ifng, Il17a, and Il22 genes n=13 naïve; n=11 PBS; n=14 FMT mice examined over four independent experiments. For Il6 and Il1b genes n=10 naïve; n=8 PBS; n=10 FMT mice examined over three independent experi-ments. Statistical significance was calculated by a two-sided unpaired t-test. *p<0.05, **p< 0.01. Data are presented as mean values ± SEM. b.d. – below detection.

Supplementary Figure 2

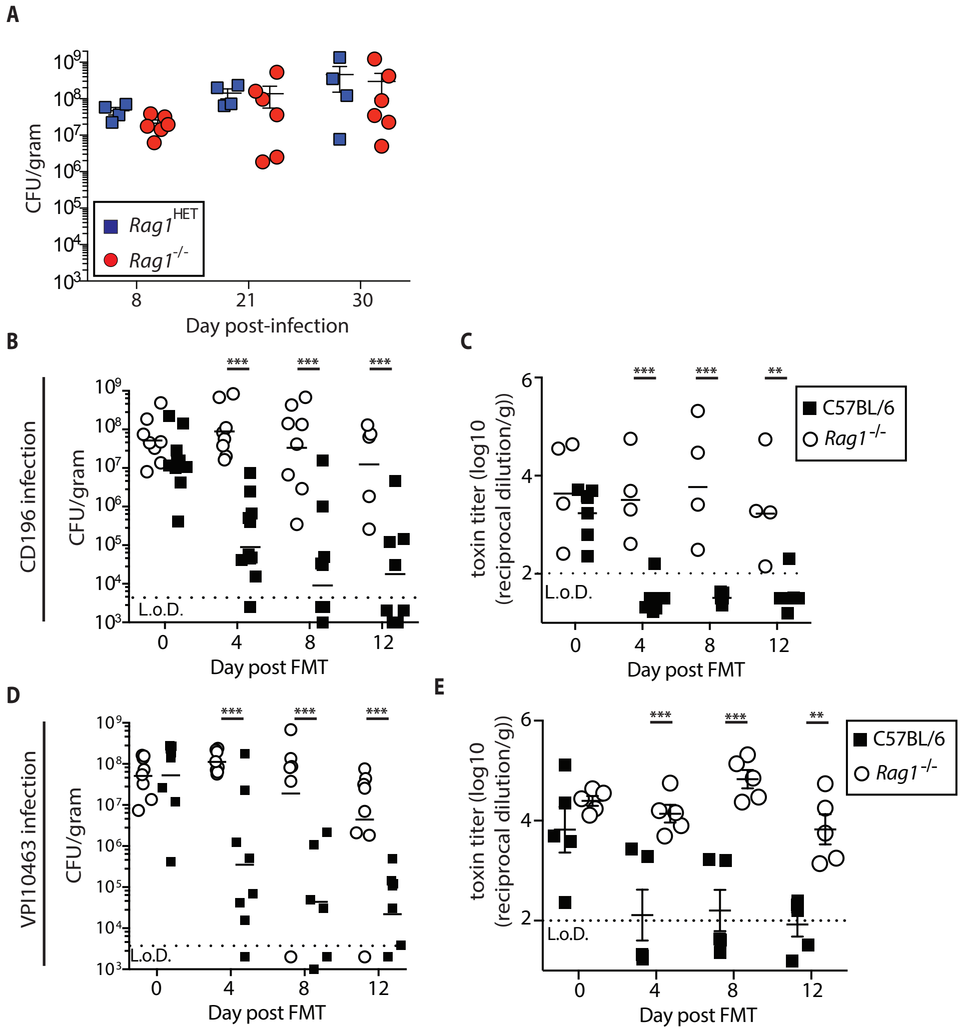

Supplementary Figure 2. Immunodeficient Rag1-/- mice exhibit impaired resolu-tion of C. difficile infection compared to cohoused C57BL/6 mice. (A) Fecal C. difficile burden following infection in littermate antibiotic-treated RagHET (n=4) and Rag1-/- mice (n=6). Data are representative of five independent experiments and are presented as mean values ± SEM. (B-E) Cohoused Rag1-/- and C57BL/6 mice were antibiotic-treated and infected with C. difficile (B-C) CD196 strain or (D-E) VPI10463 strain. (B) C. difficile bacterial burden and (C) toxin titers in fecal pellets in CD196 infect-ed Rag1-/- and C57BL/6 mice following FMT administration. (D) C. difficile bacterial burden and (E) toxin titers in fecal pellets in VPI10463 infected Rag1-/- and C57BL/6 mice following FMT administration. For data in B, n=8 Rag1-/-; n=10 C57BL/6 mice examined over three independent experiments. Statistical significance was calculated by a two-way ANOVA. ***p<0.0001 For data in C, n=4 Rag1-/-; n=6 C57BL/6 mice. Statis-tical significance was calculated by two-way ANOVA. ***p<0.0001, **p=0.007. For data in D, n=8 Rag1-/- and C57BL/6 mice examined over two independent experiments. Statistical significance was calculated by two-way ANOVA. ***p<0.001. For data in E, n=5 Rag1-/- and C57BL/6 mice. Statistical significance was calculated by two-way ANOVA. ***p<0.0005, **p=0.001. Data are presented as mean values ± SEM.

Supplementary Figure 3

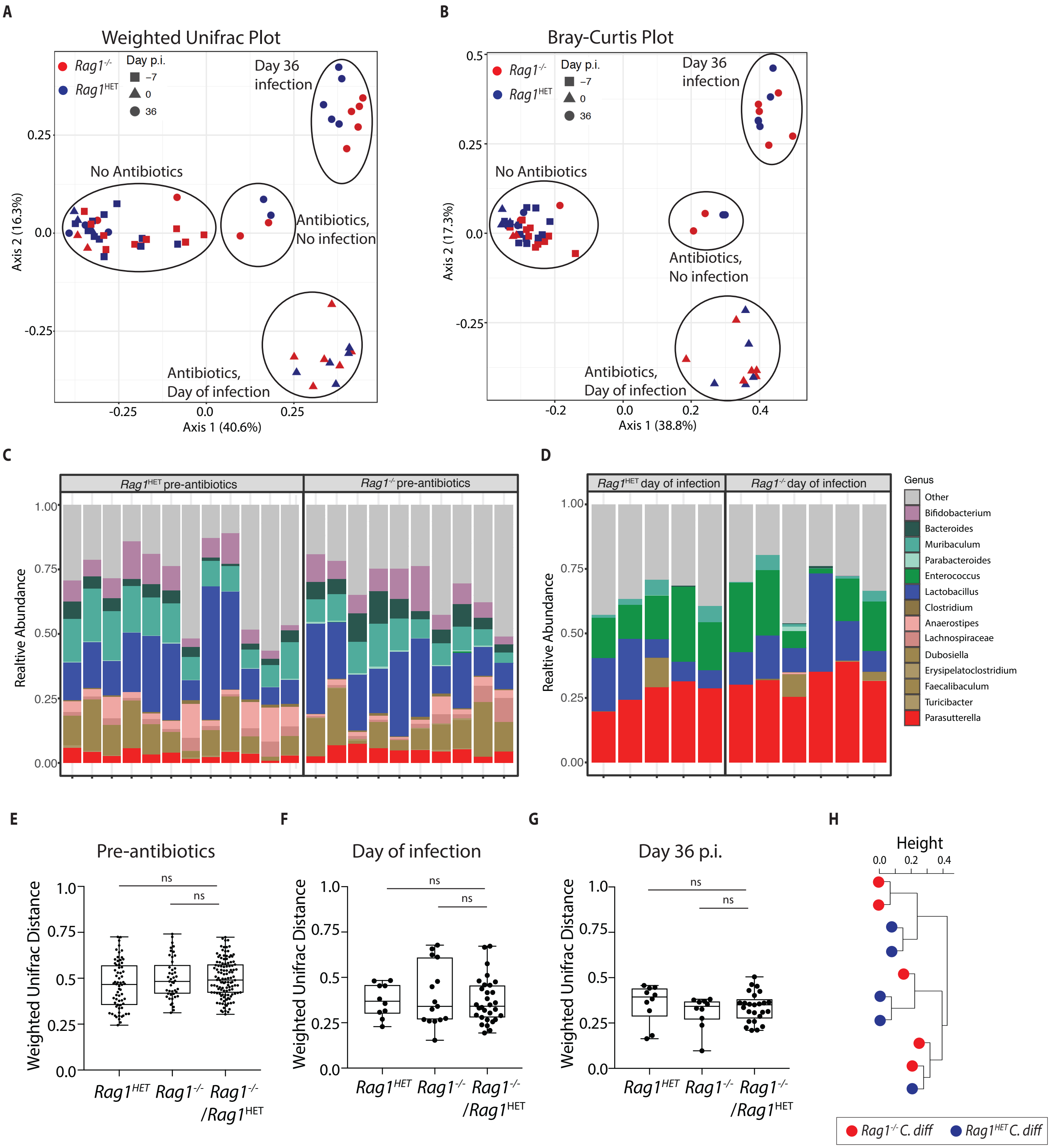

Supplementary Figure 3 Microbiota comparisons of RagHET and Rag1-/- mice. (A) Weighted UniFrac and (B) Bray-Curtis principal coordinate analysis plot of 16S bacterial rRNA ASVs from fecal pellets of Rag1-/- and Rag1HET mice prior to ABX treatment (n=10 Rag1-/-; n=12 Rag1HET mice), day 0 of infection (n=6 Rag1-/-; n=5 Rag1HET mice), or day 36 post-C. difficile (n=5 Rag1-/- and Rag1HETmice) or mock infection (n=2). Ellipses signify different experimental groups. (C-D) Relative abun-dance of top 15 bacterial ASVs in the microbiota of Rag1-/- and Rag1HET mice (C) prior to antibiotic treatment (D) and at day 0 of infection. (E-G) Weighted UniFrac distance comparing the microbiota beta diversity dissimilarity within and between Rag1HET and Rag1-/- mice (E) prior to antibiotics (n=10 Rag1-/-; n=12 Rag1HET mice), (F) at day 0 of infection (n=6 Rag1-/-; n=5 Rag1HET mice), and (G) at day 36 p.i. (n=5 Rag1-/- and Rag1HETmice). One-way ANOVA conducted for statistical comparison using Dunnett method for multiple comparison adjustments. Boxes represent median, first and third quartile. Whiskers extend to the highest and lowest data point. (H) Dendrogram representation of intestinal microbial communities of C. difficile-infected of Rag1-/- and Rag1HET mice using unsupervised hierarchical clustering of weighted UniFrac distances to identify similarities between samples.

Supplementary Figure 4

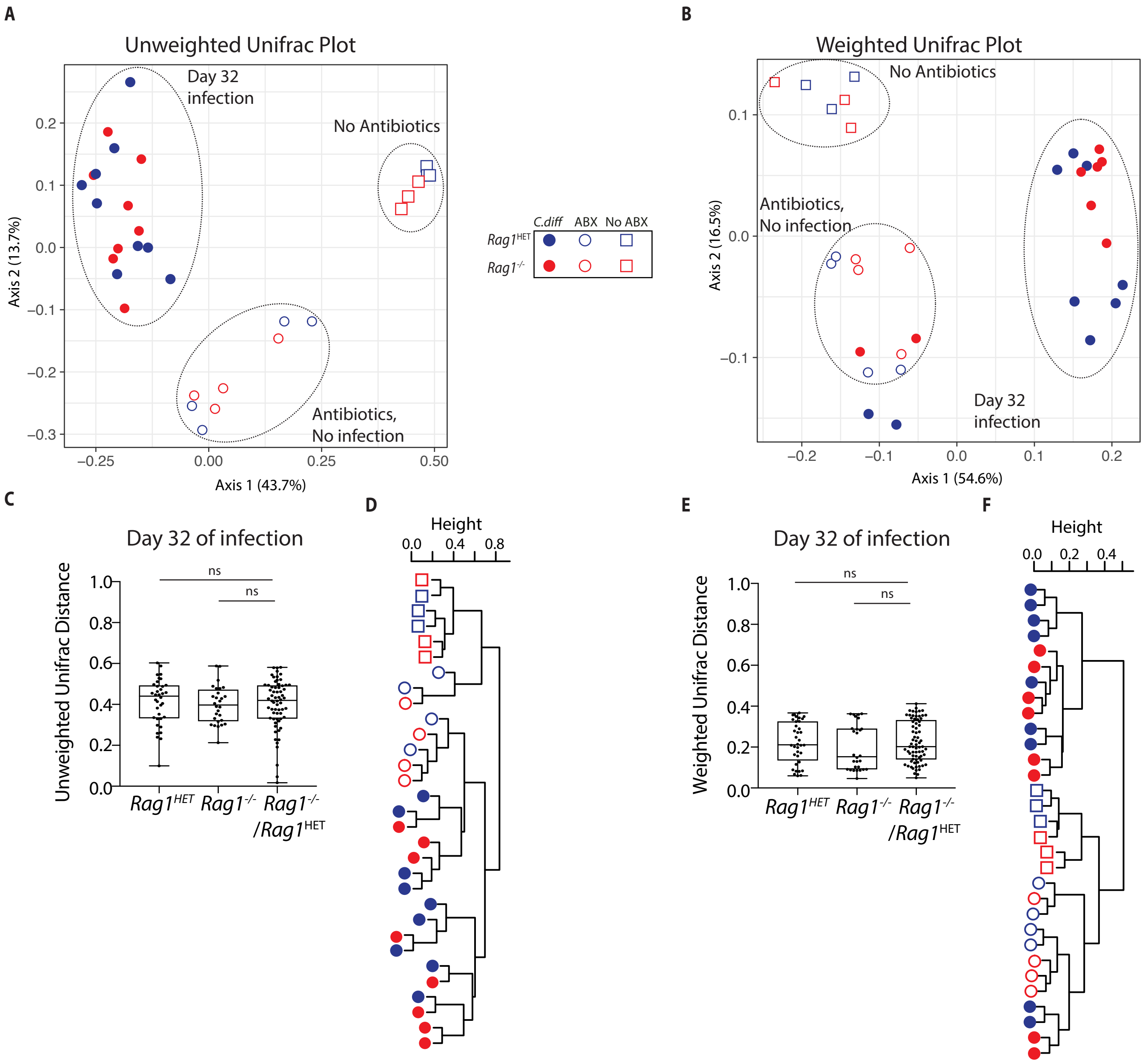

Supplementary Figure 4 Microbiota comparisons of RagHET and Rag1-/- mice at time of FMT in validation cohort. (A) Unweighted and (B) weighted UniFrac principal coordinate analysis plot of 16S bacterial rRNA ASVs from fecal pellets of Rag1-/- and Rag1HET mice at day 32 post C. difficile infection (day of FMT, Rag1HET, n= 9; Rag1-/-, n=8 mice), day 32 post antibiotic treatment alone (n=4) and day 32 no antibiotic treatment (n=3). Ellipses signify differ-ent experimental groups. (C) Unweighted and (E) weighted UniFrac distance comparing the microbiota beta diversi-ty dissimilarity within and between Rag1HET (n= 9) and Rag1-/- (n=8) mice at day 32 post C. difficile infection. One-way ANOVA conducted for statistical comparison using Dunnett method for multiple comparison adjustments. Boxes represent median, first and third quartile. Whiskers extend to the highest and lowest data point. (D,F) Dendro-gram representation of intestinal microbial communities of C. difficile-infected of Rag1-/- and Rag1HET mice using unsupervised hierarchical clustering of (D) unweighted and (F) weighted UniFrac distances to identify similarities between samples.

Supplementary Figure 5

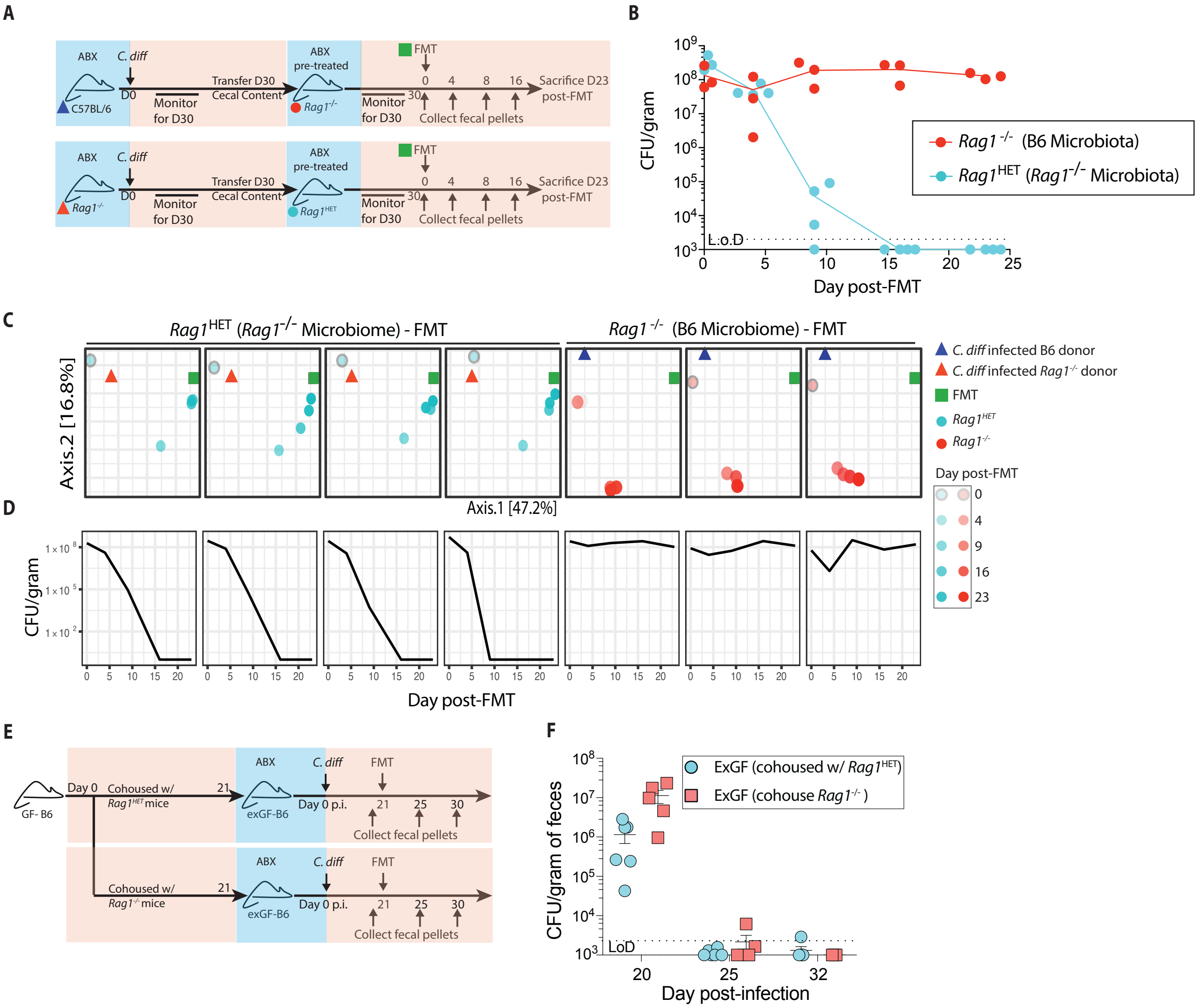

Supplementary Figure 5. Rag1-/- mice transplanted with a microbiota derived from C. difficile-infected C57BL/6 mice fail to resolve C. difficile following FMT. (A) Experimental schematic. Antibiotic-treated C57BL/6 and Rag1-/- were infected with C. difficile. At day 30 p.i. cecal content containing C. difficile was trans-ferred into reciprocal antibiotic-treated Rag1-/- (n=3) or littermate Rag1HET mice (n=4). At day 21 post cecal transplant, mice were administered FMT and (B) C. difficile burden monitored in the fecal pellets. (C) UniFrac principal coordinate analysis of 16S bacterial rRNA sequence reads from the fecal pellets of mice post FMT. Each PCoA plot represents a timecourse of an individual mouse. Green squares represent FMT source. Red or blue triangles represent the original microbiota donor. (D) Corresponding plots of C. difficile burden in each individual mouse following FMT. Data is repesentative of two independent experiments. (E) Experimental schematic. Germ-free (GF) C57BL/6 mice were cohoused with either Rag1-/- or littermate Rag1HET mice and subsequently treated with antibiotics, infected with C. difficile and administered a FMT. (F) C. difficile burden in the fecal pellets of Ex-GF mice cohoused with Rag1-/- mice (n=5) and Ex-GF mice cohoused with Rag1HET mice (n=6) following FMT. Data are presented as mean values ± SEM.

Supplementary Figure 6

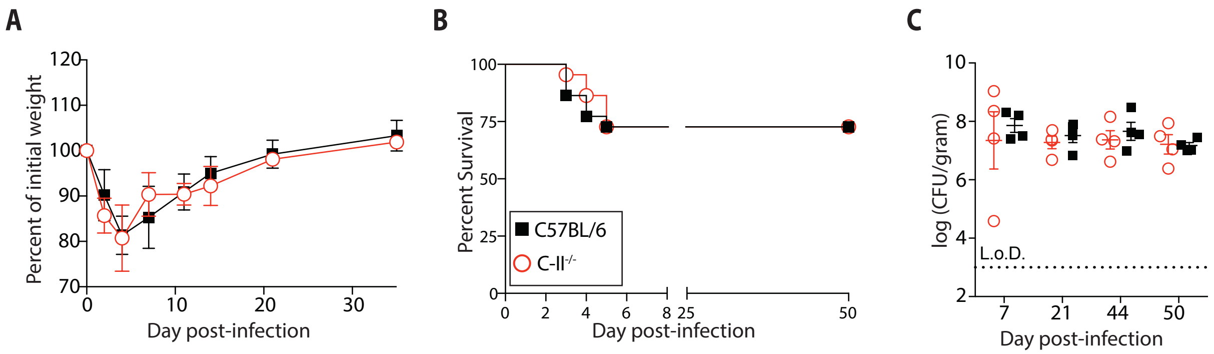

Supplementary Figure 6. CD4+ T cell deficient mice exhibit comparable acute morbidity to C57BL/6 mice and establish persistent C. difficile infection. (A) Weight loss, (B) survival, and (C) fecal C. difficile burden following infec-tion in cohoused, antibiotic-treated C57BL/6 and C-II-/- mice. Data in A and C is representative of three independent experiments. n = 4. Data are presented as mean values ± SEM. Data in B is a combination of three independent experi-ments. (C57BL/6 n=11) and (C-II-/- mice n=14).

Supplementary Figure 7

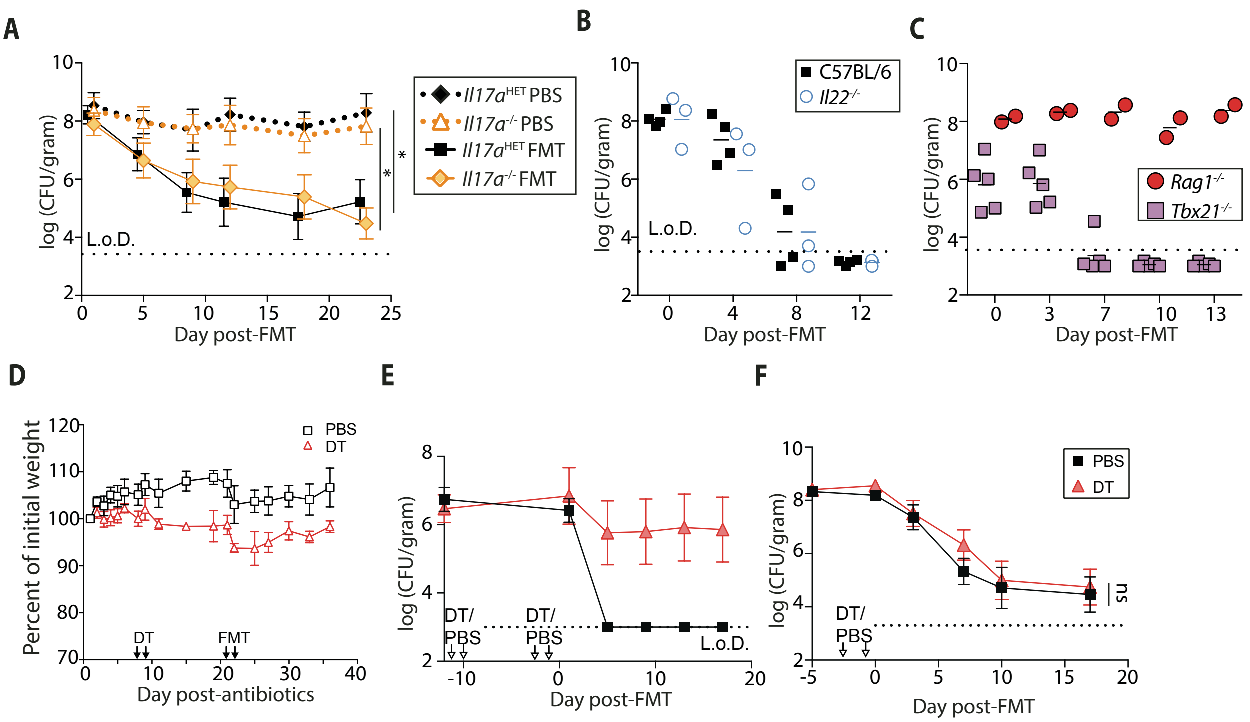

Supplementary Figure 7. TH17 and TH1 cytokines are dispensable for FMT-mediated resolution of C. difficile infec-tion. (A) Littermate Il17aHET and Il17a-/- mice were treated with antibiotics, infected with C. difficile and administered FMT or PBS at day +30 post-infection. Fecal C. difficile burden following FMT or PBS administration. FMT Il17aHET (n=6), PBS Il17aHET (n=3), FMT Il17a-/- (n=5) PBS Il17a-/- (n=3). Data is representative of two independent experiments. Statis-tical significance was calculated by a two-tailed unpaired t-test. (FMT Il17aHET vs. PBS Il17aHET *p=0.022; FMT Il17a-/- vs. PBS Il17a-/- *p=0.011). Data are presented as mean values ± SEM. (B) Cohoused C57BL/6 (n=4) and Il22-/- (n=3) mice were treated with antibiotics, infected with C. difficile and administered FMT at day +30 post-infection. Fecal C. difficile burden following FMT. (C) Cohoused Tbx21-/- (n=4) and Rag1-/- (n=2) mice were treated with antibiotics, infected with C. difficile and administered FMT at day +30 post-infection and fecal C. difficile burden was assessed following FMT. (D) Weight loss of ABX-treated, uninfected Foxp3DTR mice following DT or PBS administration. n = 3 mice. Data is represen-tative of three independent experiments. (E,F) Foxp3DTR mice were treated with antibiotics, infected with C. difficile and administered diphtheria toxin (DT) or PBS (E) 12, 11, 2 and 1 days prior to FMT or (F) 2 and 1 days prior to FMT. Fecal C. difficile burden following FMT or PBS administration. (E) DT.FMT (n=4), PBS.FMT (n=2). (F) DT.FMT (n=7), DT.PBS (n=4) mice. Statistical significance was calculated by two-way ANOVA. . Data are presented as mean values ± SEM. * = $p < 0.05$, *** = $p < 0.001$.

Supplementary Figure 8

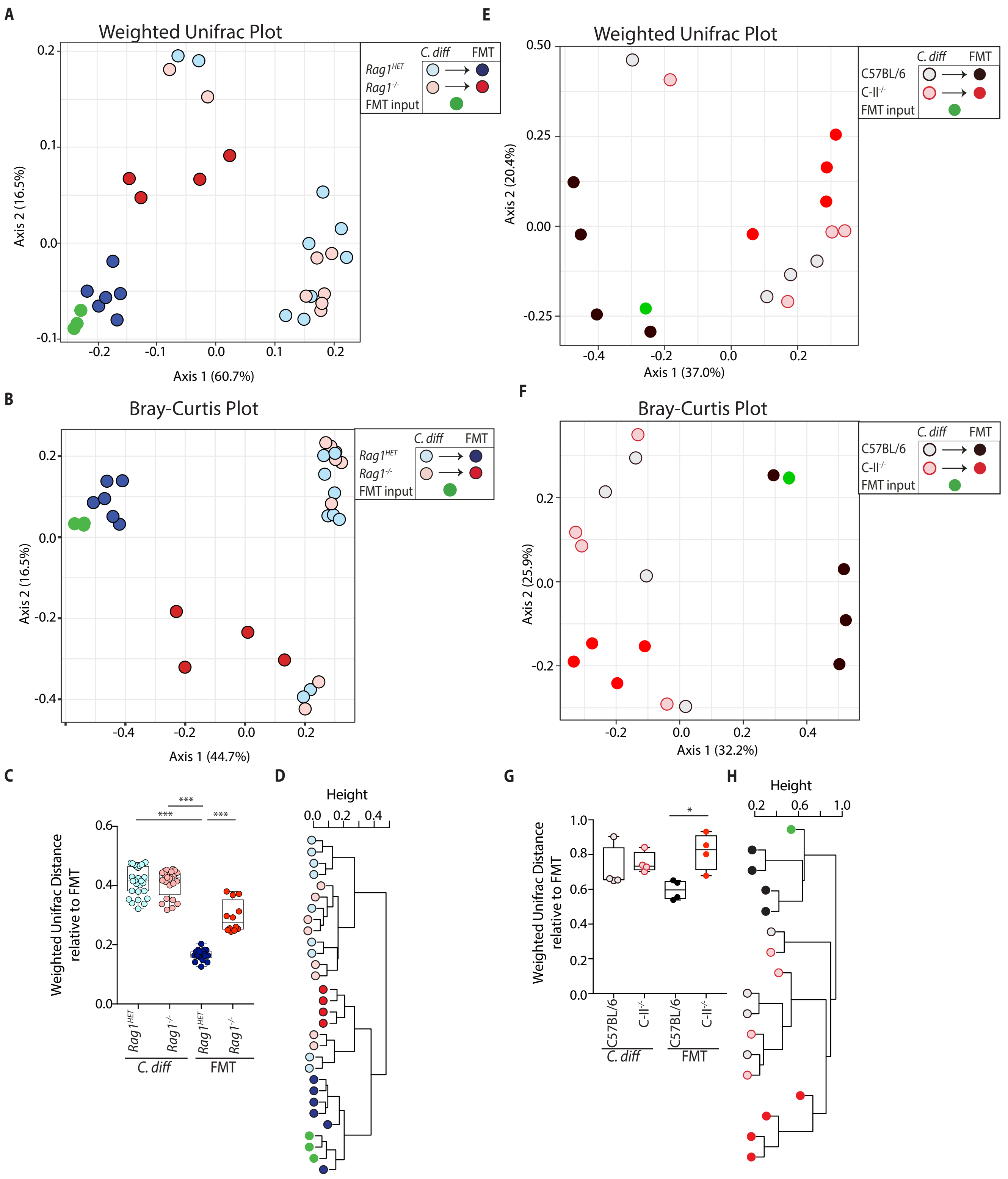

Supplementary Figure 8. C. difficile-infected Rag1-/- and C-II-/- mice exhibit impaired FMT engraftment. (A-D) C. difficile-infected Rag1-/- and Rag1HET mice or (E-H) C57BL/6 and C-II-/- mice were administered FMT and microbial composition was analyzed. (A) Weighted UniFrac and (B) Bray-Curtis principal coordinate analysis plot of 16S bacterial rRNA ASVs from the FMT inoculum, fecal pellets of C. difficile infected Rag1-/-and Rag1HET mice at the time of FMT (day 32 p.i.), and cecal content of Rag1-/- and Rag1HET mice at day 21 post-FMT. (C) Microbiota beta diver-sity dissimilarity comparing Rag1-/- or Rag1HET groups to FMT inoculum using weighted UniFrac distance. n=9 C. diff. Rag1HET; n=8 C. diff. Rag1-/-; n=6 FMT Rag1HET; n=4 FMT Rag1-/- mice. Statistical significance was calculated by a one-way ANOVA test using Dunnett method for multiple comparison adjustments. ***p<0.001. Boxes represent median, first and third quartile. Whiskers extend to the highest and lowest data point. (D) Dendrogram representation of intestinal microbial communities of FMT inoculum, Rag1-/- and Rag1HET groups using unsupervised hierarchical clustering of weighted UniFrac distances to identify similarities between samples. (E) Weighted UniFrac and (F) Bray-Curtis principal coordinate analysis plot of 16S bacterial rRNA ASVs from the FMT inoculum and fecal pellets of C. difficile infected C57BL/6 and C-II-/- mice at time of FMT and day 15 post-FMT. (G) Microbiota beta diversity dissimilarity comparing C57BL/6 or C-II-/- groups to FMT inoculum using weight-ed UniFrac distance. n=4 C. diff. C57BL/6, C. diff. C-II-/-, FMT C57BL/6, and FMT C-II-/-mice. Statistical significance was calculated by a one-way ANOVA test using Dunnett method for multiple comparison adjustments. *p=0.024. Boxes represent median, first and third quartile. Whiskers extend to the highest and lowest data point. (H) Dendrogram representation of intestinal microbial communities of FMT inoculum, C57BL/6 or C-II-/- groups using unsupervised hierarchical clustering of weighted UniFrac [illegible]

Supplementary Figure 9

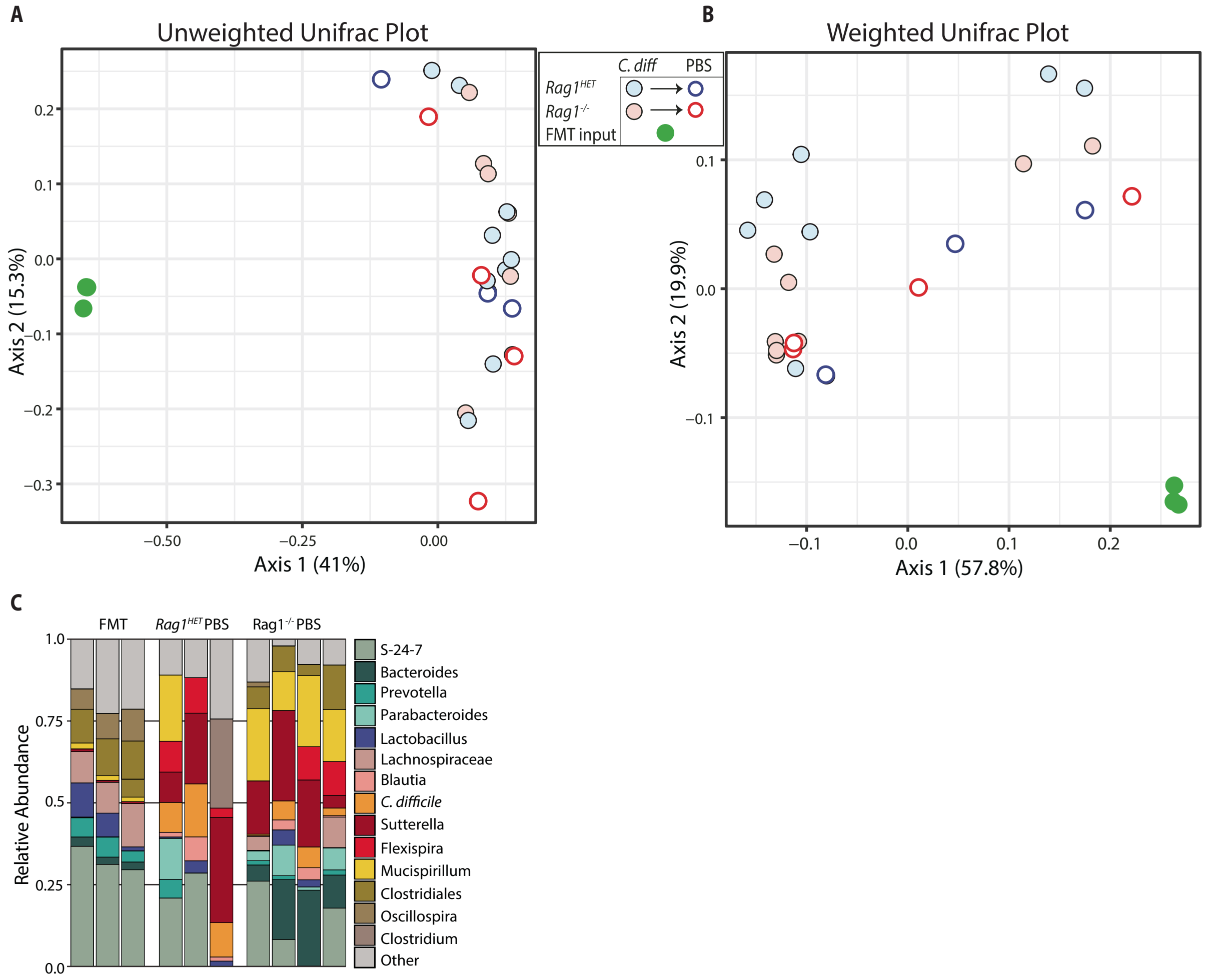

Supplementary Figure 9. PBS treatment of C. difficile-infected Rag1-/- and Rag1HET mice does not alter the microbiota. (A-C) C. difficile-infected Rag1-/- and Rag1HET mice were administered PBS and microbial composition was analyzed. (A) Unweighted and (B) weighted UniFrac principal coordinate analysis plot of 16S bacterial rRNA ASVs from the FMT inoculum, fecal pellets of C. difficile infected Rag1-/- and Rag1HET mice at the time of PBS (day 32 p.i.), and cecal content of Rag1-/- and Rag1HET mice at day 21 post-PBS treatment. n=9 C. diff. Rag1HET; n=8 C. diff. Rag1-/-; n=3 PBS Rag1HET; n=4 PBS Rag1-/- mice. (C) Relative abundance of top 15 bacterial ASVs in the microbiota of C. difficile-infected PBS-treated Rag1-/- and Rag1HET mice (day 21 post-PBS) compared to FMT inoculum. Bar plot is displayed at the genus level except for orange bars that represent an ASV aligning to C. difficile.

Supplementary Figure 10

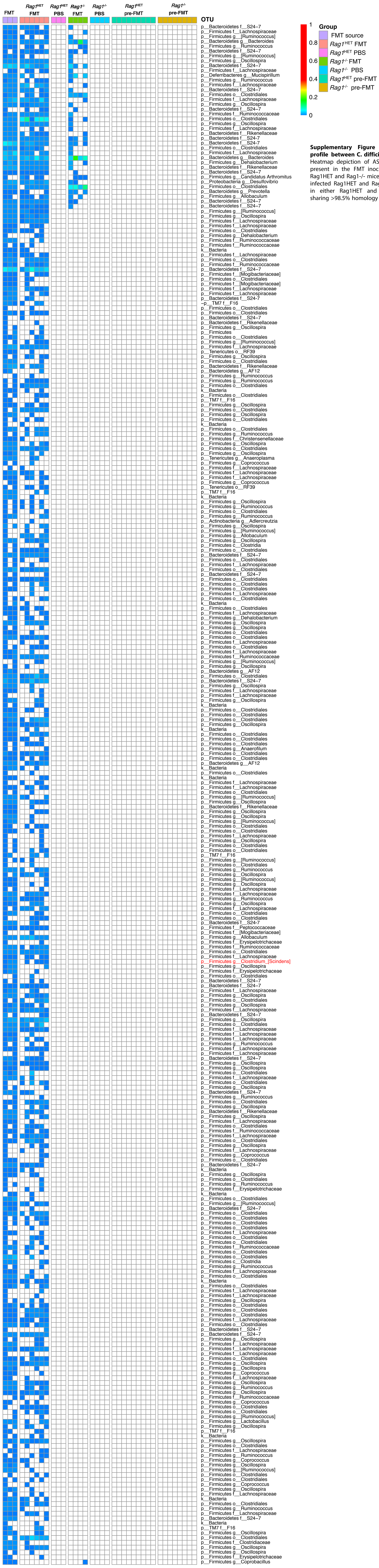

Supplementary Figure 10. Distinct bacterial engraftment profile between C. difficile infected Rag1HET and Rag1-/- mice. Heatmap depiction of ASVs that meet the following criteria (1) present in the FMT inoculum, (2) absent in C. difficile infected Rag1HET and Rag1-/- mice prior to FMT, (3) absent in C. difficile infected Rag1HET and Rag1-/- mice treated with PBS, (4) present in either Rag1HET and Rag1-/- mice treated with FMT. ASV sharing >98.5% homology to C. scindens is highlighted in red.

Supplementary Figure 11

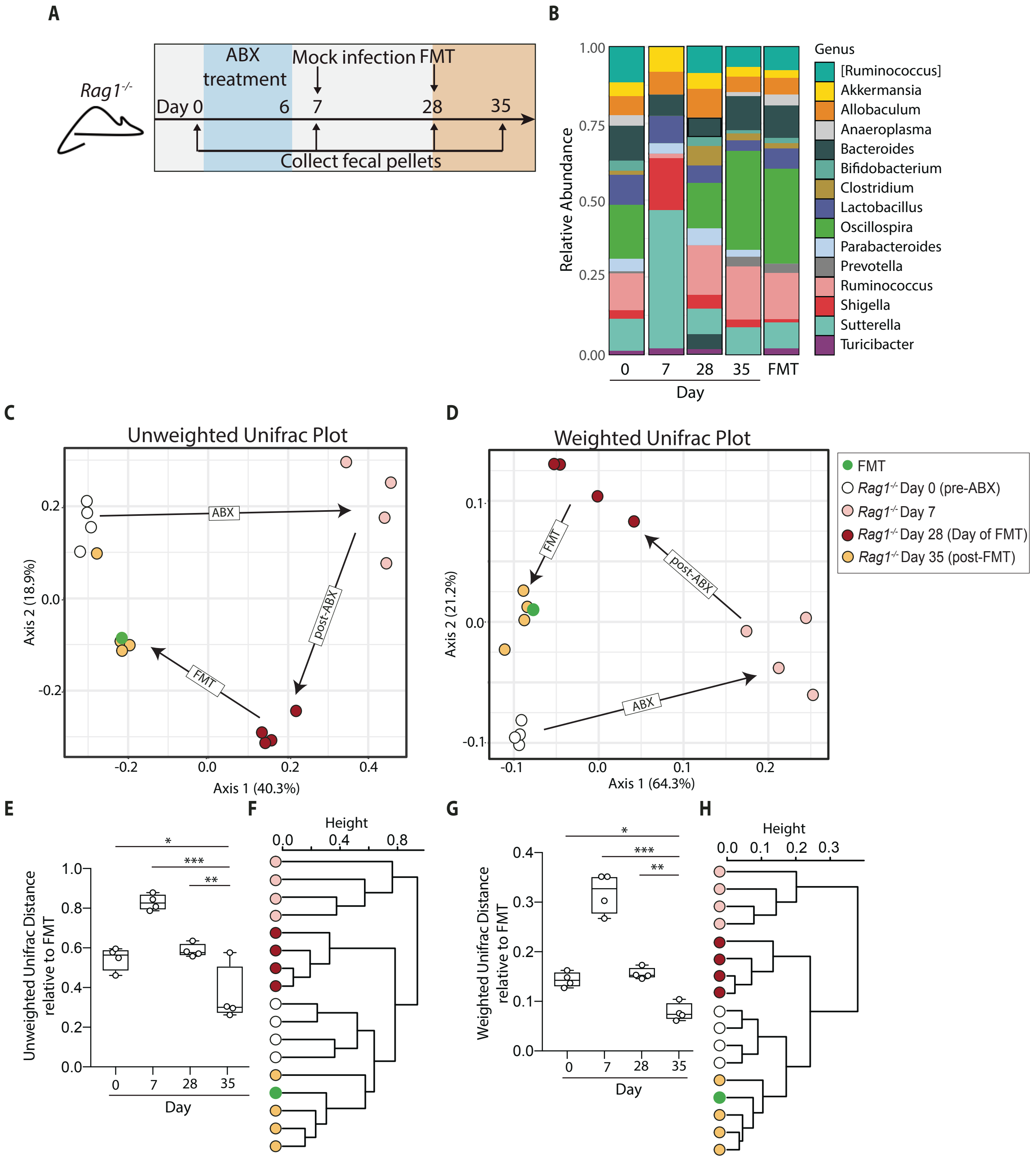

Supplementary Figure 11. Antibiotic-treated, uninfected mice Rag1-/- mice do not exhibit impaired FMT engraftment. (A) Experi-mental schematic. Rag1-/- mice were treated with the same antibiotic regimen used to predispose mice to C. difficile infection but were left uninfected and rested for 22 days following cessation of antibiotics. Mice were administered a FMT at day 28 post the start of antibi-otics. Fecal pellets were collected prior to antibiotics (day 0), immediately following antibiotics (day 7), the day of FMT (day 28), and 7 days following FMT (day 35) for 16S rRNA bacterial gene profiling. (B) Relative abundance of top 15 bacterial ASVs in the microbiota of antibiotic-treated, uninfected Rag1-/- mice. (C) Unweighted and (D) weighted UniFrac principal coordinate analysis plot of 16S bacterial rRNA ASVs over the course of the experiment. (E) Microbiota beta diversity dissimilarity comparing Rag1-/- groups to FMT inoculum using unweighted UniFrac distance. n=4 Rag1-/- mice per timepoint. Statistical significance was calculated by one-way ANOVA test using Dunnett method for multiple comparison adjustments. *p=0.014, **p=0.0031, ***p<0.0001. Boxes represent median, first and third quartile. Whiskers extend to the highest and lowest data point. (F) Dendrogram representation of intestinal microbial communities of FMT inoculum and Rag1-/- groups using unsupervised hierarchical clustering of unweighted UniFrac distances to identify similarities between samples. (G) Microbiota beta diversity dissimilarity comparing Rag1-/- groups to FMT inoculum using weighted UniFrac distance. n=4 Rag1-/- mice per timepoint. Statistical significance was calculated by one-way ANOVA test using Dunnett method for multiple comparison adjustments. *p=0.019, **p=0.0059, ***p<0.0001. Boxes represent median, first and third quartile. Whiskers extend to the highest and lowest data point. (H) Dendrogram representation of intestinal microbial communities of FMT inoculum and Rag1-/- groups using unsupervised hierarchical clustering of weighted UniFrac distances to identify similarities between samples.

Supplementary Figure 12

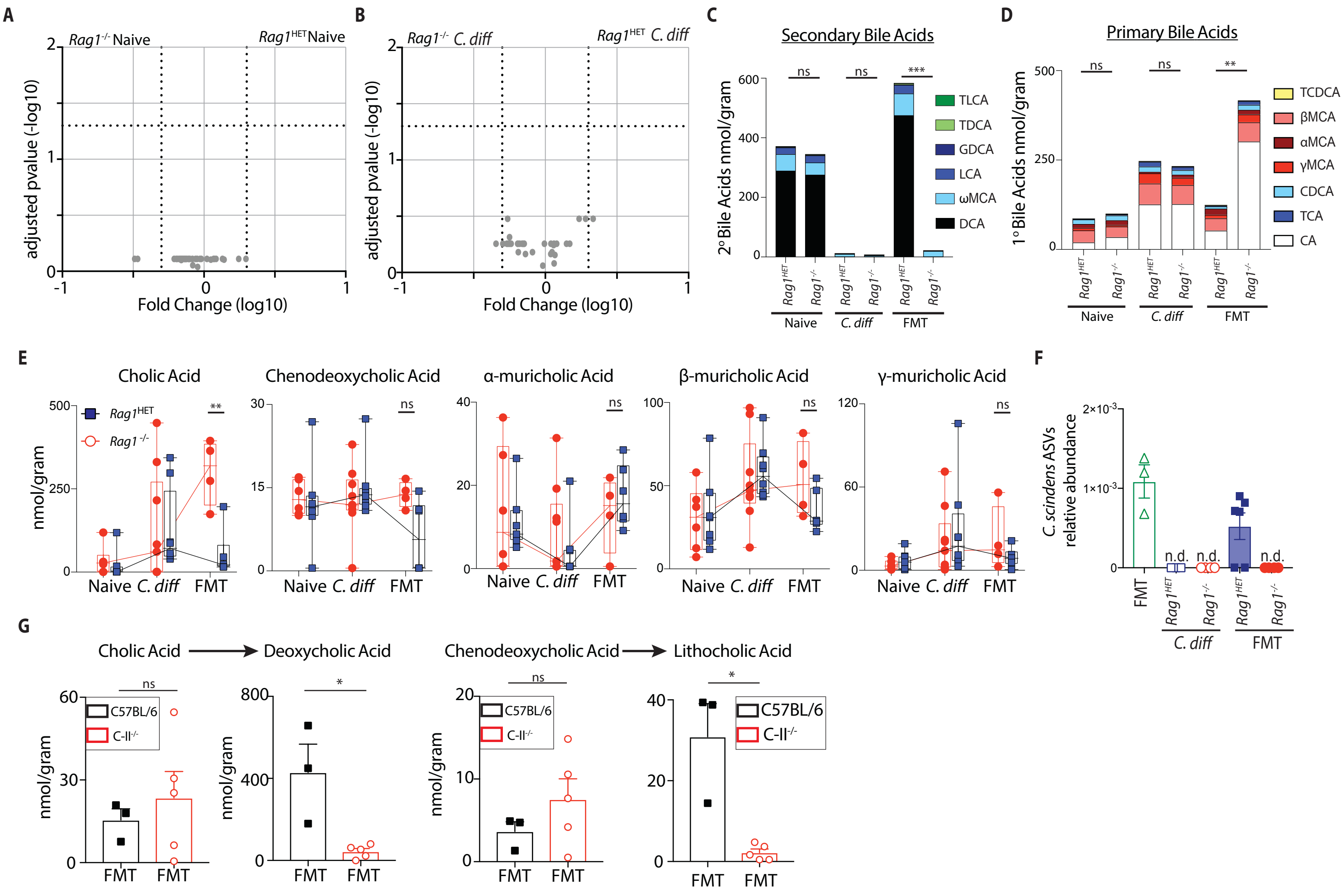

Supplementary Figure 12. FMT non-responsive mice fail to restore secondary bile acid levels. C. difficile-infected Rag1-/- and Rag1HET mice were administered FMT or PBS, sacrificed 21 days later along with naïve mice and amino acid, short chain fatty acid (SCFA), 1o and 2o bile acid pools were analyzed in cecal content. Volcano plot of metabolites in the cecum of (A) naïve and (B) C. difficile-infected Rag1-/- and Rag1HET mice. Significance threshold criteria set at a two-fold change in concentration and adjusted p-value of 0.05 using an unpaired t-test and adjusted for false discovery rate. Cumulative concentration of (C) 2o and (D) 1o bile acids. Statistical significance was calculated by a two-sided unpaired t-test. **p=0.0027, ***p<0.0001. (TCDCA- taurochenodeoxycholic acid, αMCA- alphamuricholic acid, βMCA- betamuricholic acid, γMCA- gammamuricholic acid, CDCA- chenodeoxycholic acid, TCA- taurocholic acid, CA- cholic acid, TLCA- taurolithocholic acid, TDCA- taurodeoxycholic acid, GDCA- glycodeoxycholic acid, LCA- lithocholic acid, ωMCA- omegamuricholic acid, DCA- deoxycholic acid). (E) Concentration of individual 1o bile acids. n=5 naïve Rag1-/- and Rag1HET mice; n=6 C. difficile-infected Rag1-/- and Rag1HET mice; n=4 C. difficile-infected FMT-treated Rag1-/-; n=6 C. difficile-infected FMT-treated Rag1HET mice examined over two independent experiments. Statistical significance was calculated by a one-way ANOVA using Dunnett method for multiple comparison adjustments. DCA **p=0.0052. Boxes represent median, first and third quartile. Whiskers extend to the highest and lowest data point. (F) Frequency of ASVs that share > 98.5% sequence homology to C. scindens as determined by 16S rRNA sequence reads from FMT inoculum, or cecal content of C. difficile-infected or FMT-treated Rag1-/- and Rag1HET mice. n=3 FMT; n=3 C.diff Rag1HET; n=4 C.diff Rag1-/-; n=4 FMT Rag1-/-; n=6 FMT Rag1HET mice. n.d. – not detected. Data are presented as mean values ± SEM. (G) Concentration of 1o bile acids (cholic acid and chenodeoxycholic acid) and 2o bile acids (deoxycholic acid and lithocholic acid) in the cecal content of C57BL/6 (n=3) and C-II-/- (n=5) mice at day 28 post-FMT. Data is representative of 2 independent experiments. Statistical significance was calculated by a two-sided unpaired Mann-Whitney test. DCA, *p=0.036; LCA, *p=0.036. Data are presented as mean values ± SEM. b.d. – below detection.

Supplementary Figure 13

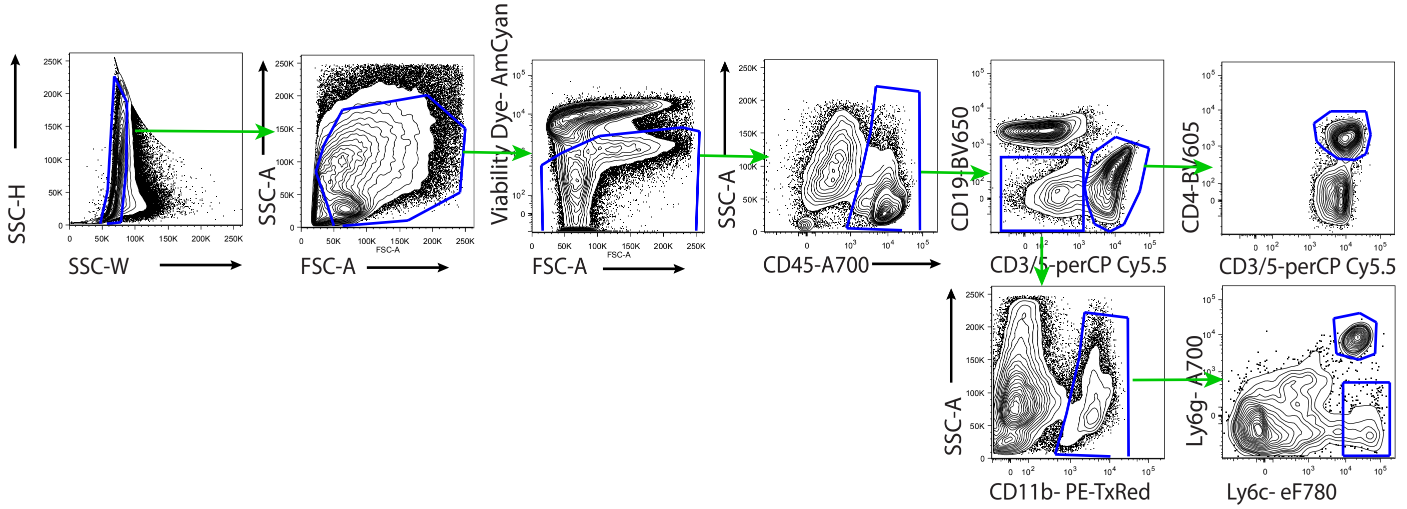

Supplementary Figure 13. Flow Cytometry Gating Strategy. Gating strategy used to identify CD4 T cells, inflammatory monocytes and neutrophils in the large intestine lamina propria. Gating strategy related to data in Figure 3 & 4.

SupplementalryTable 1

PERMANOVA - Unweighted UniFrac

Pair Comparison	R^2	p-value	FDR	Significance
Rag1^{HET} vs *Rag1*$^{-/-}$ pre-ABX	0.0814	0.016	0.048	*
Rag1^{HET} vs *Rag1*$^{-/-}$ Day 0 p.i.	0.0524	0.488	0.488	ns
Rag1^{HET} vs *Rag1*$^{-/-}$ Day 36 p.i.	0.1088	0.078	0.117	ns

PERMANOVA - Weighted UniFrac

Pair Comparison	R^2	p-value	FDR	Significance
Rag1^{HET} vs *Rag1*$^{-/-}$ pre-ABX	0.1135	0.025	0.062	ns
Rag1^{HET} vs *Rag1*$^{-/-}$ Day 0 p.i.	0.0332	0.675	0.675	ns
Rag1^{HET} vs *Rag1*$^{-/-}$ Day 36 p.i.	0.1441	0.041	0.062	ns

Supplementary Table 1. PERMANOVA analysis of unweighted and weighted UniFrac distances between the intestinal microbial communities of Rag1HET and Rag1-/- mice before antibiotic treatment, at day 0 of infection and at day 36 post C. difficile infection. The Benjamini & Hoch-berg method was used to adjust for multiple comparisons. Statistical tests performed on data displayed in Figure 2 and Supplemental Figure 3

Supplementary Table 2

PERMANOVA - Unweighted UniFrac

Pair Comparison	R^2	p-value	FDR	Significance
Rag1^{HET} vs *Rag1*$^{-/-}$ Day 32 p.i.	0.0572	0.511	0.5348	ns
Rag1^{HET} vs *Rag1*$^{-/-}$ Day 32 ABX only	0.1274	0.216	0.2430	ns
Rag1^{HET} vs *Rag1*$^{-/-}$ No ABX	0.2129	0.400	0.4286	ns

PERMANOVA - Weighted UniFrac

Pair Comparison	R^2	p-value	FDR	Significance
Rag1^{HET} vs *Rag1*$^{-/-}$ Day 32 p.i.	0.1090	0.100	0.1184	ns
Rag1^{HET} vs *Rag1*$^{-/-}$ Day 32 ABX only	0.1307	0.158	0.1777	ns
Rag1^{HET} vs *Rag1*$^{-/-}$ No ABX	0.3256	0.300	0.3293	ns

Supplementary Table 2. PERMANOVA analysis of unweighted and weighted UniFrac distances between the intestinal microbial communities of Rag1HET and Rag1-/- mice at day 32 post C. difficile infection, day 32 post antibiotic only treatment and day 32 no antibiotic treatment. The Benjamini & Hochberg method was used to adjust for multiple comparisons. Statistical tests performed on data displayed in Supplemental Figure 4.

Supplementary Table 3

PERMANOVA - Unweighted UniFrac

Pair Comparison	R^2	p-value	FDR	Significance
Rag1^{HET} FMT vs *Rag1*$^{-/-}$ FMT	0.4804	0.009	0.025	*
Rag1^{HET} FMT vs *Rag1*^{HET} *C. diff*	0.5856	0.001	0.008	**
Rag1^{HET} FMT vs *Rag1*^{HET} PBS	0.5557	0.009	0.025	*
Rag1$^{-/-}$ FMT vs *Rag1*$^{-/-}$ *C. diff*	0.3649	0.001	0.008	**
Rag1$^{-/-}$ FMT vs *Rag1*$^{-/-}$ PBS	0.3489	0.053	0.066	
Rag1^{HET} PBS vs *Rag1*^{HET} *C. diff*	0.0963	0.428	0.446	
Rag1$^{-/-}$ PBS vs *Rag1*$^{-/-}$ *C. diff*	0.1795	0.037	0.053	
Rag1^{HET} PBS vs *Rag1*$^{-/-}$ PBS	0.1348	0.489	0.489	

PERMANOVA - Weighted UniFrac

Pair Comparison	R^2	p-value	FDR	Significance
Rag1^{HET} FMT vs *Rag1*$^{-/-}$ FMT	0.5659	0.007	0.020	*
Rag1^{HET} FMT vs *Rag1*^{HET} *C. diff*	0.5753	0.001	0.009	**
Rag1^{HET} FMT vs *Rag1*^{HET} PBS	0.5461	0.009	0.025	*
Rag1$^{-/-}$ FMT vs *Rag1*$^{-/-}$ *C. diff*	0.3879	0.006	0.020	*
Rag1$^{-/-}$ FMT vs *Rag1*$^{-/-}$ PBS	0.3066	0.143	0.168	
Rag1^{HET} PBS vs *Rag1*^{HET} *C. diff*	0.1347	0.201	0.220	
Rag1$^{-/-}$ PBS vs *Rag1*$^{-/-}$ *C. diff*	0.0589	0.514	0.526	
Rag1^{HET} PBS vs *Rag1*$^{-/-}$ PBS	0.0648	0.705	0.800	

Supplementary Table 3. PERMANOVA analysis of unweighted and weighted UniFrac distances between the intestinal microbial communities of Rag1HET and Rag1-/- mice at the time of FMT and at day 21 post FMT or PBS treatment. The Benjamini & Hochberg method was used to adjust for multiple comparisons. Statistical tests performed on data displayed in Figure 5 and Supplemental Figure 8, 9.
