# Supplementary material for: Host immunity modulates the efficacy of microbiota transplantation for treatment of Clostridioides difficile infection
Source: Nat Commun. 2021 Feb 2;12:755. doi: 10.1038/s41467-020-20793-x (PMC7854624; doi:10.1038/s41467-020-20793-x)
Supplement: Supplementary file 3 — Reporting Summary [file 41467_2020_20793_MOESM3_ESM.pdf]

nature research

Corresponding author(s): Michael C. Abt

Last updated by author(s): 10/20/2020

Reporting Summary

Nature Research wishes to improve the reproducibility of the work that we publish. This form provides structure for consistency and transparency in reporting. For further information on Nature Research policies, see Authors & Referees and the Editorial Policy Checklist .

Statistics

For all statistical analyses, confirm that the following items are present in the figure legend, table legend, main text, or Methods section.

n/a	Confirmed	
☐	☒	The exact sample size (*n*) for each experimental group/condition, given as a discrete number and unit of measurement
☐	☒	A statement on whether measurements were taken from distinct samples or whether the same sample was measured repeatedly
☐	☒	The statistical test(s) used AND whether they are one- or two-sided<br>*Only common tests should be described solely by name; describe more complex techniques in the Methods section.*
☒	☐	A description of all covariates tested
☐	☒	A description of any assumptions or corrections, such as tests of normality and adjustment for multiple comparisons
☐	☒	A full description of the statistical parameters including central tendency (e.g. means) or other basic estimates (e.g. regression coefficient) AND variation (e.g. standard deviation) or associated estimates of uncertainty (e.g. confidence intervals)
☐	☒	For null hypothesis testing, the test statistic (e.g. *F*, *t*, *r*) with confidence intervals, effect sizes, degrees of freedom and *P* value noted<br>*Give P values as exact values whenever suitable.*
☒	☐	For Bayesian analysis, information on the choice of priors and Markov chain Monte Carlo settings
☒	☐	For hierarchical and complex designs, identification of the appropriate level for tests and full reporting of outcomes
☒	☐	Estimates of effect sizes (e.g. Cohen's *d*, Pearson's *r*), indicating how they were calculated

Our web collection on statistics for biologists contains articles on many of the points above.

Software and code

Policy information about availability of computer code

Data collection	Raw 16S data from experiments prior to FMT and with C-II-/- mice (Figure 2, 5E-H, Supplemental Figure 3,5, 8E-H) was quality filtered and clustered into 97% ASVs using UPARSE (USEARCH v11.0667) and ASV representatives were classified using BLAST against the NCBI refseq database. Unweighted UniFrac distances were calculated using the phyloseq (v. 1.32.0) R package using R version 3.2.1. Relative abundance and PCoA plots were made using ggplot2 (v. 3.2.0). Weighted UniFrac distances were hierarchically clustered using the R function hclust from the stats package. Sequencing data from experiments post-FMT (Figure 5A-D, Supplemental Figures 4 8A-D, 9,10,11) was processed and analyzed using the QIIME2 pipeline. DADA2 (v. 1.14.1), implemented as a QIIME2 plug-in, was used for sequence quality filtering. Taxonomic analysis was done using a Naïve Bayes classifier trained on the Greengenes 13_8 99% ASVs. For diversity metrics including UniFrac distances, a rooted phylogenetic tree was generated: first, a multiple sequence alignment was performed using MAFFT98 and high variable positions were masked to reduce noise in a resulting phylogenetic tree. A mid-point rooted tree was then generated using FastTree99.
Data analysis	Statistical analyses of non DNA sequence datasets were performed using Prism GraphPad software v6.0. All flow cytometry data was analyzed by FlowJo v 9.9.6 (Treestar). Crypt length was measured using ImageJ software (1.52q). For 16S sequencing data, in order to test the null hypothesis of no differences in the study group centroids, a PERMANOVA test as implemented by the function adonis() in the vegan package 2.5-5 [https://CRAN.R-project.org/package=vegan] was used in R 3.6.0. MetabolAnalyst 4.0Exploratory Statisical Analysis package was used to analyzed targeted metabolite screen data.

For manuscripts utilizing custom algorithms or software that are central to the research but not yet described in published literature, software must be made available to editors/reviewers. We strongly encourage code deposition in a community repository (e.g. GitHub). See the Nature Research guidelines for submitting code & software for further information.

Data

Policy information about availability of data

All manuscripts must include a data availability statement. This statement should provide the following information, where applicable:

- Accession codes, unique identifiers, or web links for publicly available datasets
- A list of figures that have associated raw data
- A description of any restrictions on data availability

Data supporting the findings of the study are available within the manuscripts and the raw values available in the source data file. For sequence data, ASV representatives were classified using BLAST against the NCBI refseq database. Sequence data that support the findings of this study have been deposited in the NCBI SRA database. Accession number PRJNA668190. (https://www.ncbi.nlm.nih.gov/bioproject/PRJNA668190).

Field-specific reporting

Please select the one below that is the best fit for your research. If you are not sure, read the appropriate sections before making your selection.

[x] Life sciences [ ] Behavioural & social sciences [ ] Ecological, evolutionary & environmental sciences

For a reference copy of the document with all sections, see nature.com/documents/nr-reporting-summary-flat.pdf

Life sciences study design

All studies must disclose on these points even when the disclosure is negative.

Sample size	In vivo mouse experiments used the number of animals in accordance with the Guidelines of the Institutional Animal Care and Use Committee (IACUC). Sample size for mouse experiments was determined by feasibility and the magnitude of the interventions impact. Some experiments had reduced mouse numbers due to humane endpoints being reach in mouse prior to FMT and start of the experiment. For FMT studies in this manuscript, the impact of FMT-mediated resolution of C. difficile infection was large, with 4-5 log fold reduction in C. difficile burden, enabling comparison groups to consist of 4 or more mice. All experiments were repeated at least two times.
Data exclusions	Data from mice that succumbed to infection prior to FMT were excluded from data analysis. These data include C. difficile burden counts.
Replication	All experiments reported in this manuscript were replicated at least once. Experiment replication is reported in the figure legend of the manuscript.
Randomization	Mice were either littermates or cohoused for at least three weeks prior to the start of the experiment. Mice were age and gender matched.
Blinding	The experimenter performing in vivo experiments was not blinded due to having designed and executed these experiments. Individuals process tissue for cytokine quantification, gene expression, toxin quantification, C. difficile burden, and bile acid quantification were blinded. Individuals computationally analyzing the 16s rDNA datasets were also blinded to the groupings.

Reporting for specific materials, systems and methods

We require information from authors about some types of materials, experimental systems and methods used in many studies. Here, indicate whether each material, system or method listed is relevant to your study. If you are not sure if a list item applies to your research, read the appropriate section before selecting a response.

Materials & experimental systems

n/a	Involved in the study	
[ ]	[x]	Antibodies
[ ]	[x]	Eukaryotic cell lines
[x]	[ ]	Palaeontology
[ ]	[x]	Animals and other organisms
[x]	[ ]	Human research participants
[x]	[ ]	Clinical data

Methods

n/a	Involved in the study	
[x]	[ ]	ChIP-seq
[ ]	[x]	Flow cytometry
[x]	[ ]	MRI-based neuroimaging

Antibodies

Antibodies used

Antibody Clone Fluorochrome Catalog# Dilution Company
CD3e 1452C11 PerCP Cy5.5 45-0031-82 1-200 eBioscience
CD4 RM4-5 BV605 100548 1-200 Biolegend
CD5 53-7.3 PerCp Cy5.5 45-0081-82 1-300 eBioscience

CD8a 53-6.7 PerCp Cy5.5 45-0081-82 1-300 eBioscience
CD19 6D5 BV650 115541 1-100 Biolegend
CD45 30-F11 BV605 103155 1-200 Biolegend
CD45 30-F11 A700 103128 1-100 Biolegend
IL17A TC11-18H10.1 FITC 506908 1-300 Biolegend
IL-22 1H8PWSR PE 12-7221-82 1-100 eBioscience
FoxP3 FJK-16S FITC 11-5773-82 1-300 eBioscience
RORgT B2D PE 12-6981-82 1-100 eBioscience
T-bet 4B10 APC 50-5825-82 1-200 eBioscience
CD11b PE TxRed RM2817 1-300 Invitrogen
Ly6g 1A8 A700 127622 1-100 Biolegend
Ly6c HK1.4 eF780 47-5932-82 1-200 eBioscience

Validation

Flow cytometry antibodies validated by manufacturer against murine cells. CD3e, CD4, CD5, CD8a, CD11b, CD19, CD45 dilution), Ly6c and Ly6g antibodies have been reported for use in surface marker staining followed by flow cytometric analysis. IL-22 and IL-17 antibodies have been reported for use in intracellular staining followed by flow cytometric analysis. RORgt, Foxp3, T-bet antibodies have been reported for use in intranuclear staining followed by flow cytometric analysis.

Eukaryotic cell lines

Policy information about cell lines

Cell line source(s)

WI-38 cells ATCC CCL-75

Authentication

Cell were identified in lab by cell morphology and sensitivity to toxin assay

Mycoplasma contamination

WI-38 cell line was not tested for mycoplasma contamination

Commonly misidentified lines
(See ICLAC register)

No commonly misidentified lines were used in this study

Animals and other organisms

Policy information about studies involving animals; ARRIVE guidelines recommended for reporting animal research

Laboratory animals

C57BL/6, Rag1-/-, µMT-/-, β2M-/-, C-II-/- (H2dlAb1-Ea), Il17a-/-, Tbx21-/- and Foxp3DTR mice were purchased from the Jackson Laboratory. Il22-/- mice were provided by R. Flavell (Yale University). All mouse strains were derived on a C57BL/6 background. Two-four month old male and female C57BL/6, Rag1HET, Rag1-/-, µMT-/-, β2M-/-, C-II-/- (H2dlAb1-Ea), Il17a-/-, Il17aHET and Foxp3DTR mice were used in the study's experiments. Three month old female Tbx21-/- and IL-22-/- mice were used in the study's experiments. All mice were bred and maintained in sterile autoclaved cages under specific pathogen-free conditions and kept on a grain-based diet (Labdiet 5053). Mice were provided autoclaved water ad libitum from water bottles. Mice were kept on 12 hour light/dark cycle in room kept at 18-23 C with 40-60% humidity.

Wild animals

No wild animals were used in this study.

Field-collected samples

No field collection samples were used in this study

Ethics oversight

All animal procedures were approved by the Institutional Animal Care and Use Committee of the Memorial Sloan Kettering Cancer Center and University of Pennsylvania.

Note that full information on the approval of the study protocol must also be provided in the manuscript.

Flow Cytometry

Plots

Confirm that:

- [x] The axis labels state the marker and fluorochrome used (e.g. CD4-FITC).
- [x] The axis scales are clearly visible. Include numbers along axes only for bottom left plot of group (a 'group' is an analysis of identical markers).
- [x] All plots are contour plots with outliers or pseudocolor plots.
- [x] A numerical value for number of cells or percentage (with statistics) is provided.

Methodology

Sample preparation

Single cell suspensions were obtained from the mouse large intestine lamina propria (Lp) by longitudinally cutting the large intestine then washing out content in PBS. Intestinal tissues were incubated at 37°C under gentle agitation in stripping buffer (PBS, 5 mM EDTA, 1 mM dithiothreitol, 4% FCS, 10µg/mL penicillin/streptomycin) for 10 minutes to remove epithelial cells followed by another 20 minutes to remove intraepithelial lymphocytes. The remaining tissue was digested with collagenase IV

1.5 mg/mL (500 U/mL), DNase 20 µg/mL in complete media (DMEM supplemented w/ 10% FBS, 10 µg/mL penicillin/ streptomycin, 50 µg/mL gentamicin, 10 mM HEPES, 0.5 mM-mercaptoethanol, 20 µg/mL L-glutamine) for 30 minutes at 37°C under gentle agitation. Supernatants containing the Lp fraction were passed through a 100m cell strainers and resuspended in 40% Percoll. Samples were then centrifuged for 20 minutes at 600x g to obtain IEL and Lp cell fractions.

Instrument	LSR II flow cytometer (Becton Dickinson)
Software	FlowJo v 9.9.6
Cell population abundance	No FACS-cell sorting was conducted in this study
Gating strategy	FACS plots gated on singlet, live, CD45+, CD3ε+, CD4+ cells. Cytokine and transcription factor population gates based on isotype controls. Monocytes and Neutrophils gating strategy was singlet, live, CD45+, Non-T Non-B, CD11b+, Ly6c+(monocytes), Ly6g+ (neutrophils)

☒ Tick this box to confirm that a figure exemplifying the gating strategy is provided in the Supplementary Information.
